# Spatial Soundscapes and Virtual Worlds: Challenges and Opportunities

**DOI:** 10.3389/fpsyg.2020.569056

**Published:** 2020-11-11

**Authors:** Chinmay Rajguru, Marianna Obrist, Gianluca Memoli

**Affiliations:** School of Engineering and Informatics, University of Sussex, Falmer, United Kingdom

**Keywords:** soundscape, virtual reality, sound perception, spatial audio, localization

## Abstract

There is increasing effort to characterize the soundscapes around us so that we can design more compelling and immersive experiences. This review paper focuses on the challenges and opportunities around sound perception, with a particular focus on spatial sound perception in a virtual reality (VR) cityscape. We review how research on temporal aspects has recently been extended to evaluating spatial factors when designing soundscapes. In particular, we discuss key findings on the human capability of localizing and distinguishing spatial sound cues for different technical setups. We highlight studies carried out in both real-world and virtual reality settings to evaluate spatial sound perception. We conclude this review by highlighting the opportunities offered by VR technology and the remaining open questions for virtual soundscape designers, especially with the advances in spatial sound stimulation.

## 1. Introduction

The term “soundscape” was introduced in the 1970s, when Schafer (1976) considered the concept of “positive soundmarks.” For many years, however, the word “soundscape” has been used to describe the recording and preservation of natural sounds. In projects like the UK Sound Map (The British Library, 2011), however, the concept has more recently evolved to include also the increasingly stimuli-rich acoustics of modern cities. According to this approach, our cities are not just reservoirs of unwanted sound (aka noise), but unexpectedly full of sounds that add to the immaterial heritage (Flesch et al., 2017).

In the soundscape approach, attention is shifted to the end users and communities within a modern city. The key difference from energy-focused descriptions (e.g., decibels) is that the different sounds present in a space are weighted by the listeners' perception.

The idea of managing soundscapes was introduced by the European Noise Directive (END) 2002/49/EC (European Commission, 2002). According to the END, unwanted sound, which has been a passively accepted aspect of Western societies since the Industrial Revolution, had now to be actively managed, even outside workplaces, to enhance citizens' well-being. The END also introduced a requirement to preserve quietness, a concept intended to have the widest possible meaning, and—at the time—left the definition to member states. After the END, planning the soundscape of future cities (i.e., the sources of sound present in a city) means not only reducing the intensity of sources labeled as noisy, but also considering positive sounds (Payne et al., 2009). While reducing the intensity of unwanted sounds is crucial near transport infrastructure (e.g., for houses facing a busy road where the impact on health may be severe), positive sounds dominate and may make a difference further from the road, where the focus is on self-reported well-being (Memoli and Licitra, 2012; Andringa et al., 2013; Aletta et al., 2019).

How to plan these soundscape changes has been addressed by the scientific community in two ways (Kang and Schulte-Fortkamp, 2016): (1) by evaluating in local communities physical indicators closely related to perception (Licitra et al., 2005; Memoli et al., 2008a; Kang et al., 2019) and (2) by standardizing acoustic surveys such that local residents are directly questioned to assess their perceptions of and expectations for the local acoustic climate; see Fields et al. (2001) and the ISO 12913 series (ISO/TC 43/SC 1 Noise, 2018). Armed with novel indicators, in the years following the END, different researchers—from the early days (Memoli et al., 2008b; Payne et al., 2009) to the most recent (Hong et al., 2020; Oberman et al., 2020) —have introduced a future where sounds can be added to an existing urban acoustic environment to change the perception of listeners.

This led to a number of studies where the soundscape of a place (and changes to it) is evaluated remotely in a laboratory through an immersive experience designed to recreate the acoustical feeling of “being there.” Brambilla and Maffei (2010) pioneered this type of study, comparing their findings for two Italian cities (Rome and Naples) with laboratory experiences using 2D pictures and audio recordings. Similarly, Oberman et al. (2020) designed an auralization room for “virtual soundwalks” in three typologically unique cities (Graz, Zagreb, and Zadar), each with perceptible soundmarks (e.g., the Sea Organ in Zadar), using ambisonic audio (through loudspeakers) and 360° pictures on a screen.

This process, which in this work we will call “remote soundscape assessment,” is also commonly used by global architecture firms. Arup's SoundLab (Forsyth, 2018), for instance, is an anechoic room where ambisonic audio is delivered through 12 speakers surrounding the listener. This setup allows Arup to evaluate the effects of noise action plans and has recently been used to evaluate Heathrow's updated respite procedure.

However, all these studies, which are often at the frontier between sonic art and immersive experiences, describe interventions on either the temporal aspect of an existing soundscape (e.g., whether we can add to a place a sequence of sounds that will be perceived as pleasant) or its frequency content (e.g., whether we can add sounds in a specific frequency range to alter perception). Moreover, they highlight the limits of just considering the temporal aspect, since multiple “non-acoustical” parameters can affect the judgments of a soundscape. These include the expectations of the listener (Miller, 2013; Sung et al., 2016; Aletta et al., 2018), and also the specific location, the local urban design and its visual appearance, the type of activities that happen there, and the listener's age, culture, and personal history (Kang and Schulte-Fortkamp, 2016).

In this mini-review, we address the often neglected impact on perception of where the sounds (appear to) come from in a remote assessment of soundscapes. Very little is, in fact, known about building and characterizing soundscapes from a spatial point of view. Even when spatialization is part of the soundscape design process, as described in the review by Hong et al. (2017), the typical conclusion is: take ambisonic recordings and deliver them through headphones.

Here, we explore alternative delivery methods. In section 2, we review works where sounds with a spatial connotation have been added to a visual stimulus to increase immersivity ^1^. We mention cases using virtual reality (VR), augmented reality (AR), or mixed reality. These are different stages of a reality–virtuality continuum (Milgram and Takemura, 1994) of visual-based experiences produced by interactive displays, typically head-mounted. Experiences where sound is delivered either by loudspeakers or by headphones.

In section 3, we describe a selection of the increasing number of studies that use VR to evaluate potential changes to existing soundscapes. section 5 summarizes research on one critical aspect of soundscape design for VR: finding the optimal number of sources needed to maximize immersivity. In particular, we discuss studies that identify: (1) how close two sources can be for the listener to distinguish them, (2) the minimal number of sources to achieve a desired localization accuracy, and (3) the relation between localization accuracy and perceived immersivity. Section 6 summarizes our findings and highlights the unanswered questions.

Note that our analysis is limited, as it neglects the theory behind sound perception. The reader can find more about this subject and the success of 3D audio elsewhere (e.g., Begault, 2000; Hong et al., 2017; Roginska and Geluso, 2018).

## 2. Virtual Reality: A Tool for Evaluating Sound Perception

Sound within human interfaces has not been developed anywhere near the level that visual interfaces have. Light-based special effects are common and light-based holograms are so well-known that they can be used as cheap souvenirs. However, as any theater or cinema director knows, achieving this level of control with sound is expensive. It requires either a large number of speakers or for everyone in the audience to wear headphones.

In VR, however, this level of control is more readily available. Moreover, audio is crucial for situational awareness. As summarized by Yung and Khoo-Lattimore (2019), the success of an immersive virtual environment (IVE) is based on three key elements: (1) the ability to look around (visualization), (2) a suspension of belief and physical representation of objects (immersion), and (3) a degree of control over the experience (interactivity). As reported by Hruby (2019) in a review on virtual cartography, different experiments support the positive impact of sound on spatial presence in IVEs. That is, sound is crucial for a user's feeling of being there. As an example, Kern and Ellermeier (2020) conducted a study where participants (*N* = 36) were asked to wear noise-canceling headphones and tasked to take a stroll in a VR park while walking on a treadmill in the real world. Different sounds were fed through the headphones. The conditions *steps with background* and *background sounds* scored more than 60% on the scales *presence* and *realism*, whereas *no-headphones, noise-canceling*, and *steps only* were around 40%.

Another example is virtual tourism. Since travelers are already happy to escape into alternate realities (e.g., theme parks), it is not surprising that a multitude of tourism-focused VR utilities are emerging. Games, educational tools, destination marketing, and virtual visits to cultural heritage sites aim to deliver selected visual, audio, and most importantly, spatial aspects of the destination without actually being there (Yung and Khoo-Lattimore, 2019). Virtual environments like *Second Life* (Linden Lab, 2020) have also gained momentum, especially when real travel is not possible (e.g., for someone confined at home to prevent the spread of disease). In these cases, adding audio to a 360° visualization may make the experience of a virtual visit almost indistinguishable from the real one (Wagler and Hanus, 2018).

**Figure 1:**
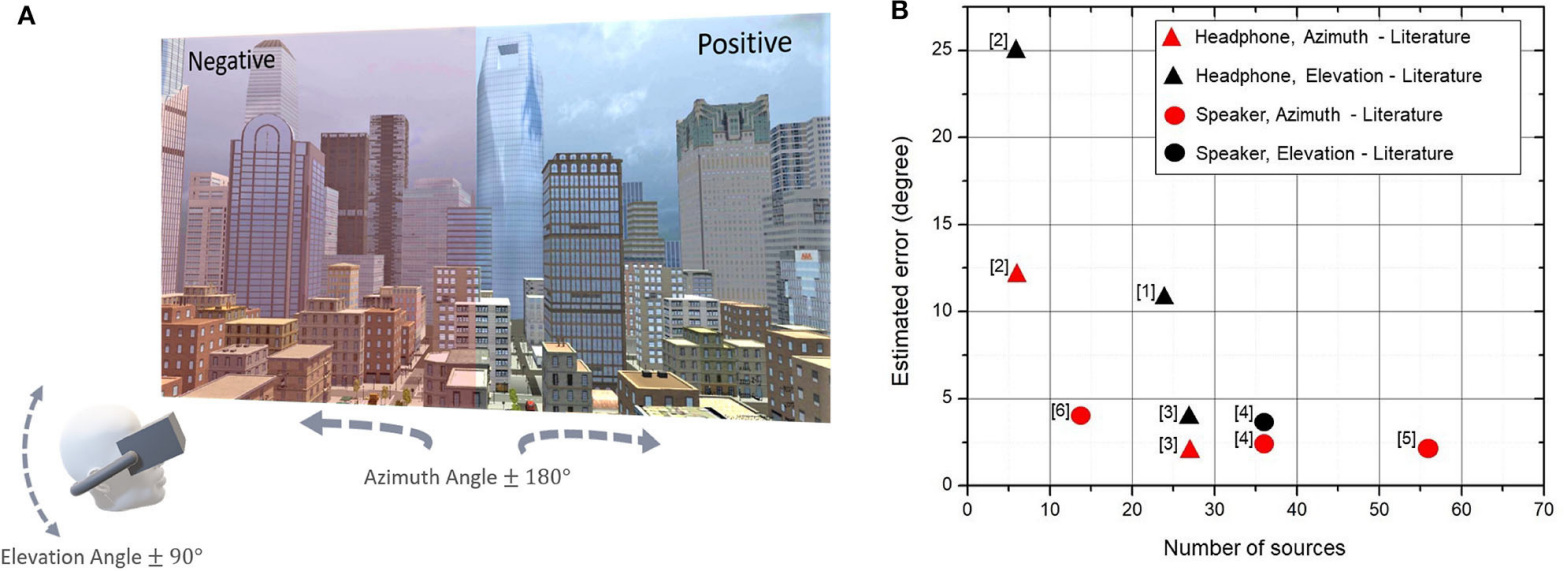
**(A)** An example of how VR could be used to evaluate changes in a specific soundscape (e.g., due to works), highlighting the role of elevation and azimuth in source localization. The 3D cityscape in this figure was created by the authors for Oculus Quest using a free city asset for Unity (Dactilardesign, 2019). **(B)** Graphical representation of estimated error found for azimuth and elevation angles from a selection of the studies in Table 1, with numbers among parenthesis referring to entries in the Table.

The above considerations show that VR is the perfect tool for perception-focused acoustic experiments (Figure 1A). Even when cross-modal interactions have been detected (Malpica et al., 2020), there seem to be a prevalence of audio over visual cues in VR. This comes from one key advantage: not only can listeners experience virtual sound sources from anywhere in a 360° space, but these can be changed as required, whereas vision requires eye or hand movements (Madole and Begault, 1995). Like what happens to blindfolded people subject to acoustic cues (Tabry et al., 2013), the success of audio in VR is, however, underpinned by the ability to localize and position where the sounds come from in the 3D (virtual) environment. It is, therefore, paramount to deliver acoustic cues so that their location is perceived accurately (section 5).

## 3. Soundscape Evaluation in VR

The low cost of VR and AR headsets and even the possibility of transforming a mobile phone into a VR visor has allowed more ambitious projects, as anticipated by Miller (2013). Lugten et al. (2018), for instance, explored the effect of adding sounds from moving water (e.g., fountains or ponds) or sounds from vegetation (e.g., sounds made by birds or the wind) in areas exposed to aircraft sounds. In their study, participants from the local community (*N* = 41) were exposed to eight different VR scenes and answered a questionnaire after each test. Lugten et al. showed that there was a marginal effect from adding only sound from vegetation, but that there was a massive positive effect for both the pleasantness and eventfulness ratings of the soundscape due to adding sound from water features or adding a combination of visual and audio aspects of water features. In particular, the eventfulness of the soundscapes increased by 26% for a sound pressure level of 70 dB and by 20% for 60 dB. Arntzen et al. (2011) went even further, with a VR-based method to asses the impact of aircraft sounds on community well-being.

Stevens et al. (2018) used VR to show how visual stimuli can alter the perception and categorization of a soundscape. In their experiments, they first presented participants (*N* = 31) with recordings taken in the North of England (in rural, urban, and suburban environments), each comprising a mixture of natural, human, and mechanical sounds. Then they presented the sounds with a visual accompaniment to represent: (1) a forest (natural), (2) a rural or suburban setting, and (3) a city center (urban). These were delivered as 360° videos through a head-mounted display (HMD). On comparing the subjective evaluations (valence, arousal, and dominance), they found a significant difference in the emotion and category ratings with and without the visuals. Again, the registered impact of vegetation was very similar to what has been found with standard surveys (Watts et al., 2011). Conversely, no significant correlation between visual and sound cues was found by Echevarria Sanchez et al. (2017). These authors used binaural sound and 360° images, delivered through a VR headset, to evaluate the renovation designs for a bridge connecting the inner city of Ghent to a large park (*N* = 75).

## 4. Soundscape Immersivity and Delivery

As shown by Jiang et al. (2018), reproducing both the visual and the audio experience is not trivial. These authors created a virtual reproduction of a Neapolitan square (Piazza Vittoria) using captured 360° images (delivered through a VR headset) and sound recordings (e.g., human voices, bird sounds, fountain sounds, sea waves, and background urban sounds) attached to corresponding objects in the VR environment. They asked participants (*N* = 100) to evaluate the IVE on 7-point scales (ranging from *poor and unrealistic* to *good and realistic*) and to leave comments about it. In this study, 62% of participants voted *good and realistic* for the visuals, but only 51% did so for the audio.

Jiang et al. (2018) concluded that, to improve the sound realism, more sound sources are needed in a VR simulation. Having more sources, however, requires more rendering power: what is gained in immersivity through the audio may be lost in the visuals. Part of the delivery could, therefore, be delegated to external real sources located around the user (e.g., loudspeakers, as in Forsyth, 2018). According to Hong et al. (2019), this may be very effective. These authors asked participants (*N* = 30) to evaluate soundscape quality and perceived spatial qualities for three spatial sound reproduction methods (static binaural, tracked binaural, and 2D octagonal speaker) and found no perceived difference. Conversely, Hruby (2019) found that loudspeakers may reduce immersivity, because headphones allow the complete exclusion of background sounds.

Choosing how the soundscape is delivered is, therefore, difficult, as there is a gap between what can be programmed (e.g., with commercial packages like Unity or Unreal) or recorded (e.g., ambisonics) and what is actually perceived by the user through arrays of speakers or headphones.

## 5. Spatial Sound: From Delivery to Perception

We are familiar with surround sound, a technology where an array of loudspeakers around the listener is used to deliver sound from 360°. Most of these setups are basically an extension of the stereo concept, where different mono audio channels are sent to each speaker and integrated in the brain of the listener. Surround sound can be found in cinemas, home theaters, and many of the soundscape studies cited earlier. In this approach, which we call fixed-speaker sources in the following (section 5.1), more loudspeakers typically lead to a more precise delivery. We also consider that Ambisonic methods are in this group. In this recording technique, first-order spherical harmonics are used to interpret the sound reaching the recording microphone so that it can be delivered into four or eight channels.

More recent methods (e.g., wave-field synthesis) try, instead, to recreate a 3D sound field physically. With 3D sound systems, it is possible to create (using interference) the field corresponding to a source positioned between two physical speakers (i.e., virtual-speaker sources in section 5.2). In modern sound bars and linear arrays, the same signal is used to feed all the loudspeakers simultaneously, with differences in amplitude and phase. These emissions combine in real space and the wavefront that reaches the listener has the right 3D information (Begault, 2000; Roginska and Geluso, 2018). However, the relation between precise delivery and the number of speakers is not straightforward.

HMDs, however, come with headphones, and this is the preferred method for delivering audio in VR and AR (see also Hong et al., 2017). In this case, the sound sources are objects placed within the simulation by a programmer. Signal processing is used to calculate what needs to be delivered to each ear of the listener, after weighting for a standardized geometry of their head (see below for head-related transfer functions). This technology, like the beamforming used in radar and medical ultrasound scanners, is typically called spatial sound and gives the listener the impression that the sound comes from all around them (Begault, 2000). Although headphone sources are all virtual, in this mini-review we distinguish two sub-categories: fixed-headphone sources where the audio object stays in a fixed position relative to the visual environment and dynamic headphone sources where the audio is linked to a movable item.

### 5.1. Delivery Using Arrays (Fixed-Speaker Sources)

In a typical experiment with speaker arrays, the loudspeakers are positioned at a fixed distance *D* from the listener, either along the azimuth or the elevation direction, and the participants experience different acoustic stimuli, apparently coming from positions on a sphere of diameter *D* (Table 1). According to Guastavino et al. (2005), however, how the sounds are recorded is crucial, as this is the first step in a process later formalized by Hong et al. (2017). Guastavino et al. (2005) captured city sounds, using either stereo or ambisonic recordings, and delivered the acoustic experience using eight fixed-speaker sources. The participants (*N* = 29 for stereo and *N* = 27 for ambisonics) were asked which setup sounded more like an everyday experience. The results showed that 2D configurations of speakers are sufficient outdoors, whereas 3D configurations should be preferred indoors.

**Table 1:** A summary of the literature considered in this mini-review on the capability of locating sound sources.

**Reference**	**Environment**	**Sound Stimuli**	**Sound Stimuli** **distance**	**Sound delivery** **method**	**No. and type** **of sources**	**No. of** **participants**	**Error found**
[1] Sodnik et al. (2006)	AR	Engine sound	min. 15 cm max. 80 cm	Headphones	24 fixed sources	10	Distance between perceived and real source <15 cm (i.e., 10.8°)
Rungta et al. (2017)	VR	Recorded human clapping	1.7 m	Headphones	7 fixed sources	17	Users overestimated distances <1 m and underestimated distances >1 m
Kose et al. (2018)	VR	Audio clip from Modern music	1.5 m	Headphones	1 dynamic source	n/a	Elevation was misjudged
[2] Yang et al. (2019)	VR	Synthesized sounds	Arbitrary distribution	Headphones	6 fixed sources	21	Mean azimuth error: 12.07° Mean elevation error: 25.06°
[3] Ahrens et al. (2019)	VR and Real	Pink noise burst	2.4 m 15° separation	27 loudspeakers	27 fixed sources: 13 along azimuth 7 each for elevation ± 28°	10	Elevation error (max. 2°) was larger when using a head mounted display (HMD)
[4] Makous and Middlebrooks (1990)	Real	System generated signals	1.2 m	36 loudspeakers	36 fixed sources	6	Azimuth error 2° Elevation error 3.5°
[5] Müller et al. (2014)	Real	Pulsed noise, speech, guitar tones	3 m	56 real loudspeakers	Multiple virtual sources	17	Azimuth error <11.5 cm (i.e., 2.2°)
Sato et al. (2020)	Real	Low-frequency noise (100 Hz 500 Hz)	1.5 m	4 real loudspeakers	Multiple virtual sources	7	Performance of judging elevation reduced after 65°
[6] Kühnle et al. (2013)	Real	Gaussian noise bursts (250 ms)	2.35 m	14 loudspeakers	14 fixed sources	136	2 ± 1° near the front 4 ± 2° at 90° from front
Litovsky et al. (2004)	Real	Pink noise bursts (170 ms, 65 dB)	1.4 m	8 loudspeakers	8 fixed sources	17	Root mean square error for bilateral signals approx. 30°

*In the typical experiment, the user is in a virtual or real environment and receives sound cue(s) from a specific distance. The stimuli are produced by a certain number of sources, located all around the user, both in the azimuth and the elevation directions. The study realizes a specific angular error in locating the sound source. References with number among parenthesis are represented graphically in the Figure 1B*.

The first parameter for characterizing these systems is the minimum audible angle (MAA), which is the smallest difference in the azimuth direction of two equal sound sources that can be reliably separated (Mills, 1958). This is assumed to be about 2° in front of the listener, which was confirmed by the experiments of Kühnle et al. (2013), who found a median value of 2.5 ± 1.1° for sources at φ ≈ 0° (14 speakers, *N* = 136 participants). The MAA, however, is thought to degrade as the angle increases, and in fact, a median of 5.3 ± 2.5° was found for sources at φ ≈ 90° (Kühnle et al., 2013). The MAA is a psychoacoustic quantity and does not seem to depend on visual stimuli (Rummukainen et al., 2020).

The second parameter is the localization accuracy, which is the maximum difference between the programmed position of the sound source and its perceived position. Although this quantity cannot be larger than the MAA, accuracy depends on the number of speakers and their positions in space. Just like changes in the output of an optical display need to be delivered quicker than the eye can perceive to produce fluid images (i.e., 0.1 s), the MAA gives acoustic designers a target for spatial accuracy.

Makous and Middlebrooks (1990), for instance, used 36 real speakers spaced at 10° intervals around a circular hoop (1.2 m radius). The participants (*N* = 6) were asked to turn their head to look in the direction of the sound while being tracked using an electromagnetic device. It was found that the performance of listeners was better in front than any other direction, with an average sound localization error of 2° in azimuth and of 3.5° in elevation.

Ahrens et al. (2019) used more speakers. They built a full sphere with 64 loudspeakers (2.4 m from the listening position) and used it to highlight the challenge of aligning the real world (i.e., the fixed loudspeakers) and the virtual world (i.e., the VR simulation) to achieve accurate spatial auditory perception. These authors used only the frontal 27 speakers and conducted tests for different user conditions, such as with or without a blindfold as well as with a HMD. With visible sources, the accuracy was very close to the MAA. However, the azimuth and elevation localization errors increased by 3° and 1.5° when the subjects were blindfolded. When participants were wearing a HMD, the azimuth error was the same but the elevation error was 2° larger. Interestingly, users performed better on the right-hand side by ≈1°.

### 5.2. Delivery Through Virtual-Speaker Sources

Müller et al. (2014) used a “BoomRoom”—a room containing a ring of 56 real loudspeakers (diameter 3 m, positioned at the ear level of the user) and 16 suspended cameras—to track a user's position and deliver virtual-speaker sources. Müller et al. (2014) created an AR experience using wave-field synthesis to create virtual sound sources originating from real objects (bottles or bowls). The participants (*N* = 17) were asked to determine the sound source location, which was accurate within 2.2° (azimuth), very close to the MAA.

More recently, Sato et al. (2020) investigated the relation between the perception of azimuth and elevation angles using only four speakers. These authors tested 40 different configurations with two acoustic signals (wideband noise: 100 Hz–20 kHz or low-pass noise: 100–500 Hz), four elevation angles (55°, 65°, 75°, and 80°), and five initial azimuth angles (0°, 45°, 90°, 180°, and −135°). The participants (*N* = 7) were asked to find the direction of the sound source (sound pressure level of 65 dB and duration of 1,600 ms) by pressing a button. The procedure was repeated 160 times. Their algorithms worked effectively when the height of the sound source was lower than 3 m and horizontally farther than 1 m. Also, 65° was the upper limit of the elevation angle.

### 5.3. Delivery Through Headphone Sources

Spatial audio uses the time and intensity differences between the signals to each ear, which underpin our ability to position a source in the horizontal plane (azimuthal angle φ = 0° to 180°, where 0° corresponds to the front of the listener) and at a certain distance (Rayleigh, 1907; Bronkhorst and Houtgast, 1999). Once the position of listener relative to the sources is known, spatial audio is relatively easy to implement. In contrast, the ability to locate a source in the vertical plane (elevation angle, θ = 0° to 90°) depends on the direction-dependent filtering of the outer ear (Roffler and Butler, 1968). There may be larger localization uncertainties for elevation, as perception depends on the individual.

In this approach, there are a few positions where the sound localization is difficult. Two obvious positions are the points just in front or just behind a user, since the signals reach each ear at the same time. To overcome this “cone of confusion” (Aggius-Vella et al., 2017), listeners simply need to move their heads, so that the angle changes until a perceivable difference is created. When the head is fixed, however, listeners hearing sounds through headphones that are processed to appear as if they come from behind them can experience localization inaccuracies as large as 45° (Steadman et al., 2019). The temporal dynamic of the source, however, is only one aspect of perception. As shown in Table 1, assumptions about the listener, the number of virtual objects producing the sound, and the amount of training received are also crucial.

#### 5.3.1. Assumptions About the User

The frequency filtering due to diffraction from the pinna, head, and torso is usually described by a head-related transfer function (HRTF) (Begault, 2000). Virtual audio systems are based on the assumption that, once the HRTF is known, any sound can be processed so that, when delivered through headphones or an array of speakers, it is perceived as coming from any desired position in 3D space (Wightman and Kistler, 1989). HRTFs are parameters of the individual, but because commercial solutions use standard functions measured with dummies (Wenzel et al., 1993), there is a potential reduction in localization accuracy (Ben-Hur et al., 2020). In addition, since HRTFs are not always recorded in non-anechoic environments, any differences between the space where the sound was recorded and the one where it is played may result in further inaccuracies, especially in terms of the perceived distance of the source, which may be as low as ≈18% of the correct distance, according to Gil-Carvajal et al. (2016).

#### 5.3.2. Source Distance and Movement

It is very challenging to estimate the distance of a source, such as the altitude of an overflying plane, and this results in large errors in real life (Memoli et al., 2018). Localization improves when a user is allowed to move towards the source, which is crucial for audio-only AR environments (e.g., a voice describing places).

Rungta et al. (2017) compared experimentally the performance of analytical algorithms (based on parametric filters) with ray tracing in rendering the distance of an acoustic source. Participants (*N* = 17) were trained blindfolded and then tasked to judge the distance to different sources, while walking along a path in VR. This study found that, although the actual distance is directly proportional to the perceived one, subjects tended to overestimate distances <1 m and underestimate distances >1 m.

Yang et al. (2019) placed everyday objects around users as spatial audio (virtual) sources in AR. These authors describe an experiment (*N* = 21) consisting of three parts. First, they delivered 3D sound through headphones, while the user was standing stationary, facing the direction of the sound. Second, they delivered sound with a visual cue (real paper boxes). For these two parts, the user was asked to find the source location while remaining in the same spot. Third, the user was allowed to walk towards the virtual sound source to identify its location. For the first part, they found a maximum azimuth localization error of 30° and an average error of 12.07°. In the second case, only 4 tests were answered incorrectly out of 168. All the users were able to find the objects accurately in the third case. Interestingly, Yang et al. (2019) noted that participants first walked in the direction of the sound to reduce the angle or distance error.

#### 5.3.3. Training

When required to localize an acoustic source, participants benefit from training. Sodnik et al. (2006), for instance, found a net improvement in performance after three attempts. Steadman et al. (2019) showed that using a game-like environment to train users can improve their ability to localize sounds. The participants (*N* = 36) listened to 19 acoustically complex stimuli positioned along a hemisphere centered on the listener. Stimuli were delivered using headphones. The tests were conducted over 3 days, during which each participant was randomly assigned to one of four groups. The control group (*N* = 9) did not receive any training while the other three groups were trained using increasing gamification elements. The perception errors for azimuth and elevation (i.e., the angular difference between where the sound was actually delivered and where it was perceived) were highly skewed, but the majority of errors for the control group were below <90°, with an average of ≈40°. All participants undergoing training had lower localization errors than the control group. The error decreased with the number of training sessions. When the participants were allowed to turn their heads, the average error was reduced to 20% after only six sessions.

#### 5.3.4. Presence of Visual Stimuli

There is often a correlation between the acoustic and visual judgments of a soundscape (Watts et al., 2011; Memoli et al., 2018; Ahrens et al., 2019). Since in VR the balance may be altered, e.g., by presenting visual and acoustic stimuli at different times, it is, therefore, important to quantify their relative weights.

Sodnik et al. (2006) highlighted the role of cross-modal correspondences using AR by delivering 3D sound through headphones. Their virtual sources were coincident with 24 identical aircraft models, randomly placed in a tabletop-sized environment (100 × 60 × 60 cm), at distances between 15 cm and 80 cm from the listener. Participants (*N* = 10) were asked to indicate a noisy object by turning their head in its direction. These authors found that the minimum distance between the head and the target was 15 cm (corresponding to a 10.8° localization error) and observed that participants performed sound localization first in the horizontal plane and then in vertical plane. They also noted that localization can be improved if there is some regularity in the distribution of the sound sources.

Kose et al. (2018) ran a similar experiment, simultaneously presenting acoustic and visual stimuli in a VR environment. They used a single speaker moving along a rectangular path. In the first part of the experiment, they localized the source using microphones and a triangulation algorithm. In the second part, they used the captured sounds to deliver 3D sound to participants in a VR environment, where the source appeared like a group of spheres. They found that the microphones often misjudged the up–down direction. Also, the users often got distracted by the delay of 500 ms due to processing.

## 6. Discussion and Conclusions

In comparing judgments on soundscapes obtained in situ with those obtained remotely [i.e., in a dedicated room with sound and visual stimuli (Brambilla and Maffei, 2010; Oberman et al., 2020)], we highlighted that only the right number of sources can recreate the auditive feeling of being there (Jiang et al., 2018), which corresponds to hearing sound all around.

As reported by Guastavino and Katz (2004), however, there is not an optimal reproduction method that works for arbitrary audio material. The choice is left to the designer, who often has only qualitative information on the differences between the delivery methods (e.g., Hong et al., 2017). Indeed, even design indications for loudness are inadequate. The few researchers who have looked into this parameter (e.g., by measuring the sound level threshold) seem to agree that the threshold does not depend on loudness (Makous and Middlebrooks, 1990; Litovsky et al., 2004; Rungta et al., 2017).

In this mini-review, we focused on one key design parameter: identifying the minimum number of sources needed for a given localization accuracy. We analyzed the literature on the spatial perception of sound and in particular the studies that use either fixed-speaker sources or fixed-headphone sources (i.e., acoustic objects fixed in a virtual landscape and delivered through headphones). In these studies—a subset of those reported in Table 1—one single speaker was active at any moment in time but, as shown in Figure 1B, we found that the localization error decreased with the number of sources. An MAA of 2° was reached in studies with at least 27 sources [13 in azimuth and 7 in ± 28° elevation angles (Ahrens et al., 2019)]. Note that Figure 1B plots the values for fixed-headphone sources and fixed-speaker sources with the same color, as they seem to follow the same trend. This suggests that to achieve a seamless spatial delivery, at least 27 sound objects are needed in a VR simulation, making the latter difficult for a portable headset. This finding needs further investigation.

Table 1 also highlights how the sound sources positioned along azimuth angles are easier to localize than sources at different elevations. This is crucial for 3D soundscape designers (Hruby, 2019) and for interpreting data in a cityscape (Memoli et al., 2018).

Once the right level of immersivity is reached, however, the soundscape evaluations obtained in VR-based experiences seem to be similar to those obtained—in the same location—from standard questionnaire surveys (Hong et al., 2017; Wagler and Hanus, 2018; Oberman et al., 2020). This observation, if confirmed by more studies, indicates that VR could be used to evaluate the impact of changes to a soundscape in urban areas, with highly reduced costs compared to field studies. Recent studies are already starting to transfer the instruments typically used for assessing real soundscapes into VR environments (Lam et al., 2020).

Before getting there, however, it is necessary to confirm whether audio in VR has the same impact as in the real world, as some of the studies we reviewed emphasize that the role of visual stimuli is at the same level observed in standard questionnaire surveys (Stevens et al., 2018; Wagler and Hanus, 2018). In contrast, other researchers report that audio is important in VR (Malpica et al., 2020) and others report no correlation between the two stimuli, at least in certain tasks (Echevarria Sanchez et al., 2017; Rummukainen et al., 2020). Equally important is to understand the positive role of training in VR, which has not been discussed in real soundscape evaluations, to date.

### 6.1. Recent Work and Future Opportunities

We are living a spring of creativity, as artists create acoustic live performances (Melody, 2020), shared spaces to meet, as well as live musical demos using combinations of virtual and real stimuli (Sra et al., 2018). One example of these mixed reality events is *Out There* (WilkinsAvenue, 2019), the first location-based immersive musical experience with a lyrical narrative. Another is the *Star Wars: Bose AR Experience* (Bose, 2019), in which users had to walk around in a physical space to explore the virtual sound and the events happening in the story. According to some authors, we will soon be able to purchase personalized soundscapes (Kiss et al., 2020). With 3D audio coming into the real world (Neoran et al., 2020), the boundaries between real and virtual are blurring.

As described in this mini-review, however, there are (potentially) large uncertainties associated with using headphones, underpinning the general consensus that external loudspeakers may create more immersive experiences, even in VR (Hruby, 2019). In addition, headphones do not allow users to comment on a shared experience in real space, reducing interactions. Further studies on alternative ways of delivering location-based sound cues—e.g., directional speakers (Yoneyama et al., 1983; Obrist et al., 2017; Ochiai et al., 2017), immersive audio domes (Ott et al., 2019), and, more recently, acoustic projectors (Rajguru et al., 2019)—would therefore be highly desirable. These methods create real sources around the listener and may, therefore, complement headphones in the virtual delivery of soundscape experiences.

## Data Availability Statement

The original contributions presented in the study are included in the article, further inquiries can be directed to the corresponding author/s.

## Author Contributions

All authors contributed equally to this manuscript. GM as an acoustician working on soundscapes, CR as an expert on virtual and augmented realities. MO as an expert in multi-sensory immersive experiences.

## Conflict of Interest

The authors declare that the research was conducted in the absence of any commercial or financial relationships that could be construed as a potential conflict of interest.

## Footnotes

^1^Often defined as the feeling of being there.
